# The State of Hepatitis B in Florida in 2025: Current Challenges and Health Disparities

**DOI:** 10.1111/jvh.70136

**Published:** 2026-01-19

**Authors:** Cameron Nejat

**Affiliations:** ^1^ Department of Public & Ecosystem Health Cornell University Ithaca New York USA; ^2^ New Jersey Medical School, Rutgers Univeristy Newark New Jersey USA

**Keywords:** elimination, epidemiology, Florida, hepatitis B, prevention, public health

## Abstract

Hepatitis B virus (HBV) infection remains a significant public health challenge in Florida despite advancements in prevention and treatment. This review analyses the current epidemiology, disease burden and management strategies for hepatitis B in Florida as of 2025, drawing on state surveillance data and recent public health reports. Florida continues to experience higher rates of both acute and chronic hepatitis B compared to national averages, with pronounced racial, ethnic and geographic disparities. Rural areas face disproportionately high rates of acute infections, while chronic infections are more concentrated in urban centres. Despite established prevention strategies—including vaccination, perinatal prevention programs and adult testing initiatives—critical gaps persist in service delivery and care engagement. This review also highlights the growing challenges posed by declining childhood immunisation rates and limited harm reduction services in high‐burden areas. Finally, we examine Florida's progress toward the World Health Organization's 2030 hepatitis elimination targets, underscoring the urgent need for expanded screening, improved linkage to care and strategies that address the social determinants driving transmission.

## 1 Introduction

Hepatitis B virus (HBV) infection represents a persistent public health challenge with significant morbidity and mortality worldwide. In the United States, in 2022, approximately 17,650 new infections occurred annually, with a case rate of 6.1 per 100,000 [1]. Despite the availability of effective vaccines and antiviral treatments, HBV continues to contribute substantially to the burden of chronic liver disease, cirrhosis and hepatocellular carcinoma [2].

Florida faces unique challenges regarding hepatitis B, with epidemiological patterns differing from national trends. As of 2023, according to the Centers for Disease Control (CDC), Florida reported an acute hepatitis B infection rate of 3.1 cases per 100,000 people, substantially higher than the national average of 0.7 cases per 100,000 [3]. Similarly, Florida's chronic hepatitis B rate of 10.1 per 100,000 far exceeds the national rate of 6.1 cases per 100,000 [1]. These disparities underscore the need for a comprehensive understanding of the current state of hepatitis B in Florida to inform targeted public health interventions.

This review aims to provide an updated assessment of hepatitis B in Florida as of 2024, examining its epidemiology, risk factors, prevention strategies, testing and treatment access and progress toward elimination goals. By identifying gaps in the current response and opportunities for improvement, this review seeks to contribute to developing more effective strategies for reducing the burden of hepatitis B in Florida.

## 2 Epidemiology of Hepatitis B in Florida

### 2.1 Incidence and Mortality Risk

**FIGURE 1:**
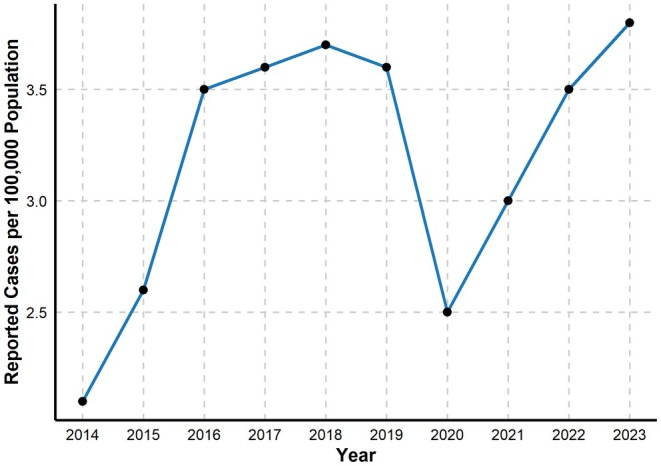
Acute hepatitis B case rates in Florida from 2014 to 2023 [4].

The epidemiological landscape of hepatitis B in Florida reveals some concerning trends in both acute and chronic infections. Between 2014 and 2023, according to the Florida Department of Health, Florida experienced a doubling of acute hepatitis B cases, rising from 408 to 866 cases, demonstrating a steady increase, as seen in Figure 1 below, despite a temporary dip during the COVID‐19 pandemic due to reduced social interactions, testing and reporting [4]. This contrasts sharply with the national trend, which showed a decline in acute HBV cases from 3218 in 2016 to 2214 in 2023, according to the CDC [5].

**FIGURE 2:**
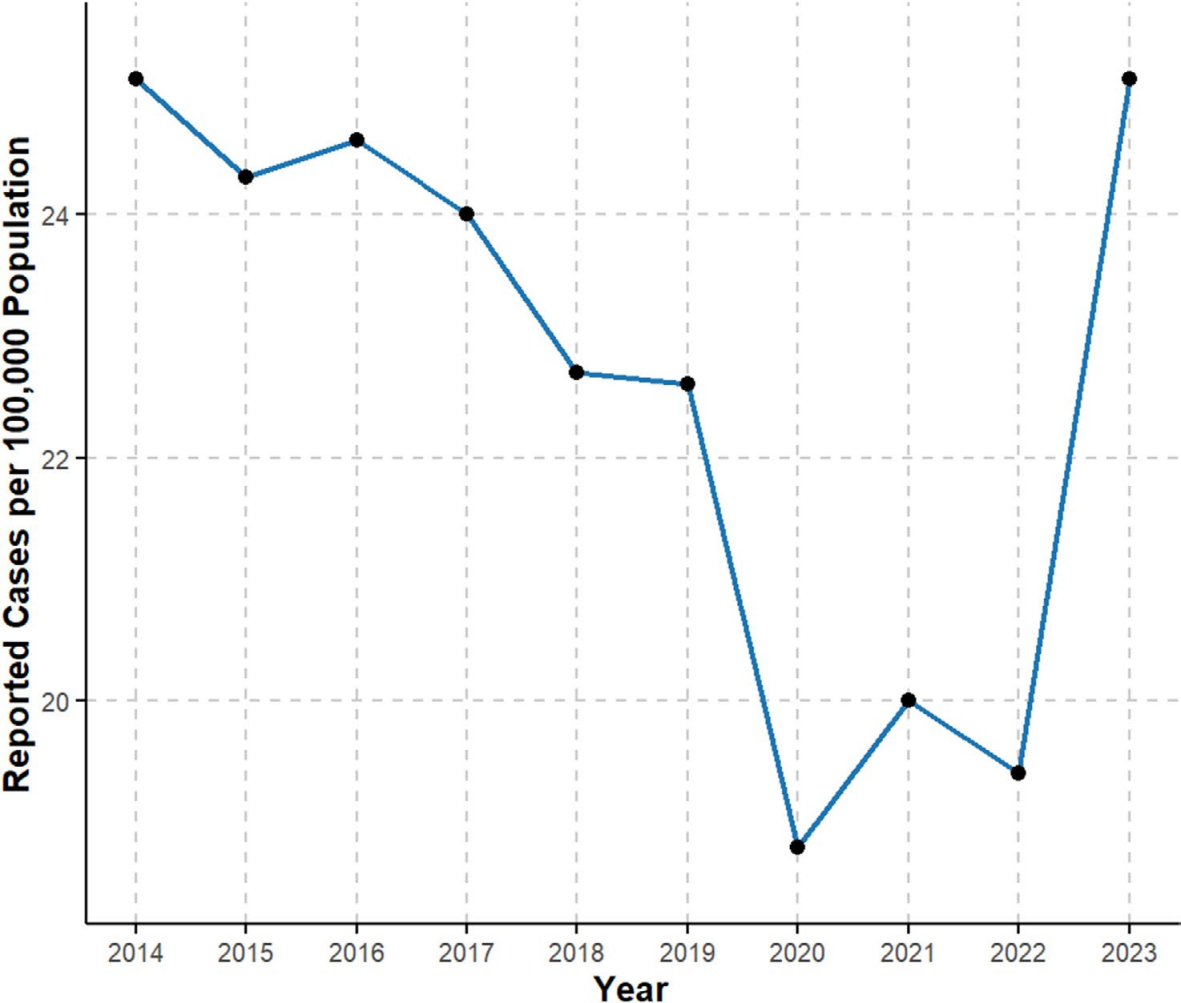
Chronic case rates hepatitis B case rates in Florida from 2014 to 2023 [6].

According to the Florida Department of Health, in 2023, Florida reported 5694 cases of chronic hepatitis B with a case rate of 25.1 per 100,000—matching the case rate from 2014, when 4914 cases were reported, as seen in Figure 2, despite a dip due to the pandemic [6]. The rise in acute cases alongside stagnant chronic case numbers indicates a lack of progress in addressing the incidence of hepatitis B in Florida.

Although Florida reports higher case rates of both acute and chronic hepatitis B compared to national averages, its mortality outcomes are slightly better. According to the CDC, Florida's hepatitis B mortality rate is 0.38 per 100,000 population, compared to the national average of 0.44 per 100,000 [7]. This suggests that, despite a higher disease burden for both acute and chronic incidence, Florida has achieved comparatively better outcomes in reducing hepatitis B‐related mortality [1, 3, 7]. Alternatively, this observed reduction may be influenced by factors unrelated to true declines in mortality, such as out‐migration of individuals with chronic HBV prior to progression to end‐stage liver disease, or potential misclassification of cause of death in vital records when underlying HBV infection is not specified and deaths are attributed instead to liver cancer or cirrhosis.

### 2.2 Demographic Distribution

Substantial demographic disparities continue to characterise the distribution of hepatitis B in Florida. According to the 2023 Integrated Epidemiological Profile from the Florida Department of Health, which reported both acute and chronic cases in Florida, case rates were highest among adults aged 35–44 at 49.2 per 100,000, followed by those aged 45–54 (45.5 per 100,000) and 55–59 (39.3 per 100,000) [8]. Gender disparities remain prominent, with males accounting for 53.9% of cases and exhibiting a higher case rate of 31.5 per 100,000, compared to 26.0 per 100,000 among females [8]. Racial and ethnic disparities also persist; Black individuals had the highest reported rate at 48.2 per 100,000, followed by Hispanic/Latino individuals at 19.0 per 100,000, while White individuals had the lowest rate at 16.4 per 100,000 [8]. Additionally, individuals classified as ‘Other’ (180 cases) and ‘Not Specified’ (1515 cases) continue to represent a disproportionately affected group, which likely reflects the high prevalence of hepatitis B among Asian and Pacific Islander populations originating from endemic regions [8, 9]. These patterns highlight the need for targeted prevention and intervention efforts in disproportionately affected populations.

### 2.3 Geographic Distribution

**FIGURE 3:**
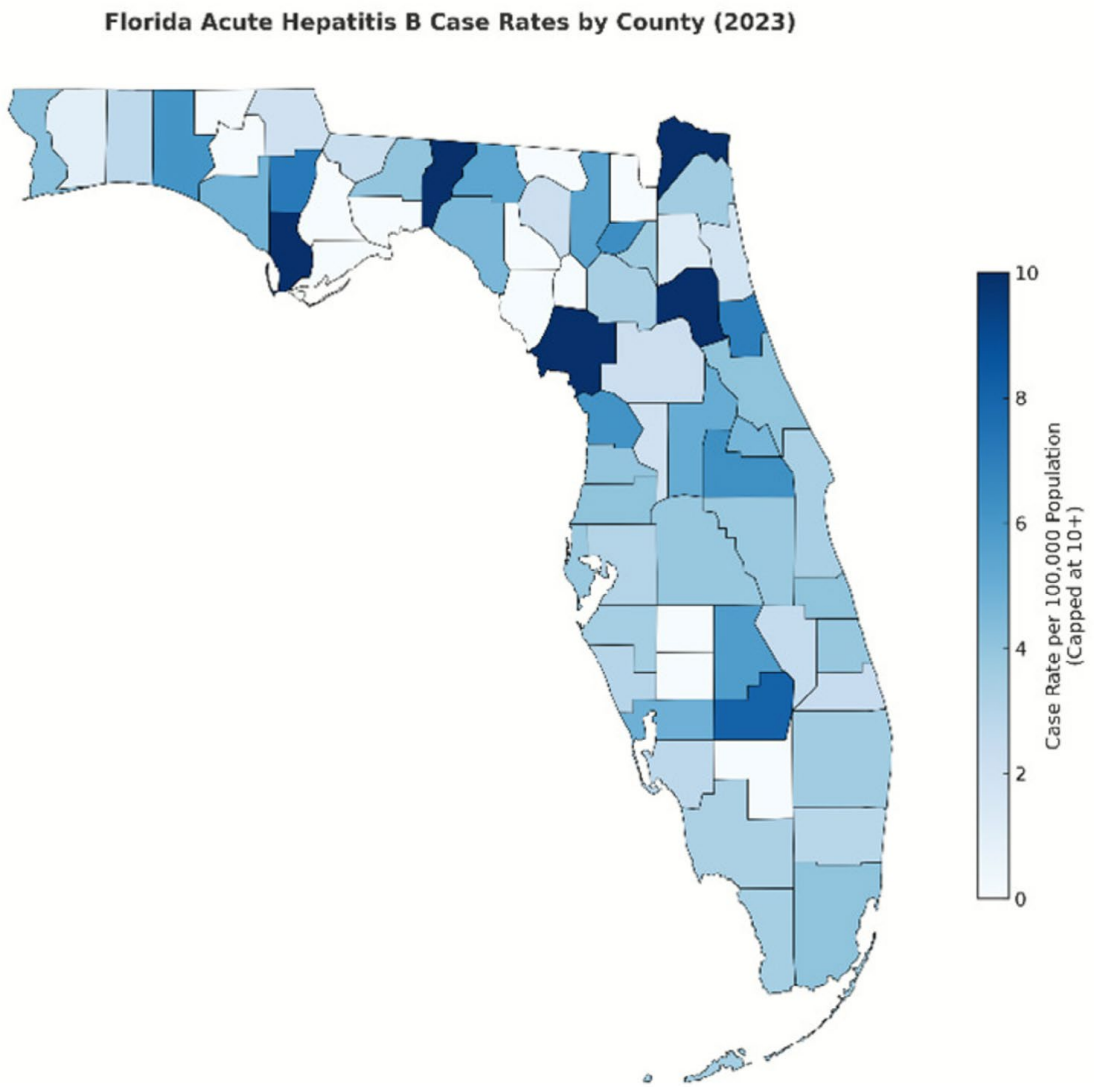
Map of acute hepatitis B distribution by county in Florida 2023 [4].

**FIGURE 4:**
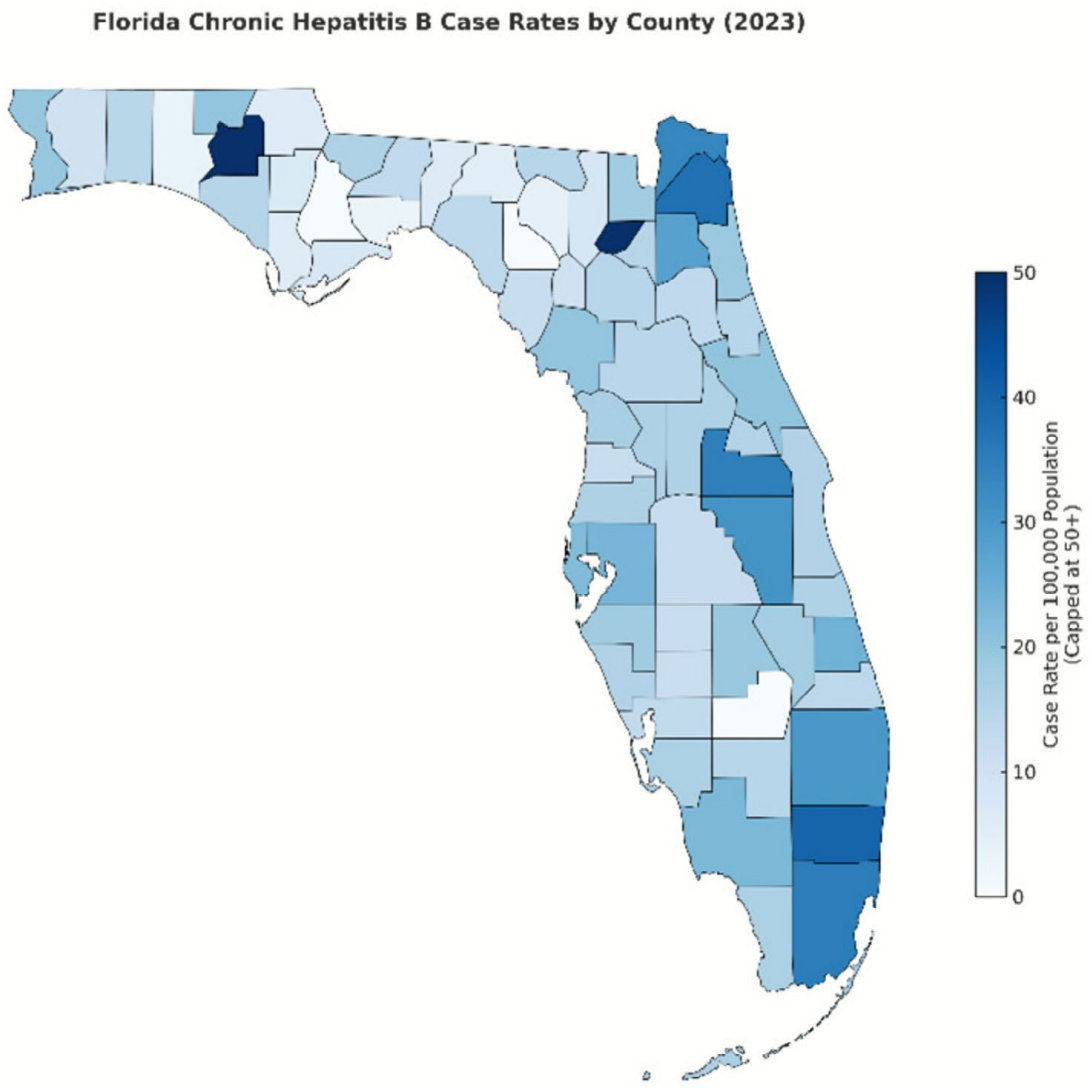
Map of chronic hepatitis B distribution by county in Florida 2023 [6].

Geographically, acute hepatitis B exhibits a distinct rural concentration, with the highest case rates per 100,000 in 2023 observed in rural counties: Jefferson (19.8), Levy (13.4), Putnam (12.1), Gulf (12.0) and Nassau (10.1), as seen in Figure 3 [4]. The distribution of chronic hepatitis B differs, with the highest rates in a mix of rural and urban counties: Washington (146.9), Union (140.2), Broward (40.0), Miami‐Dade (35.2) and Orange (34.9), as seen in Figure 4 [6]. This geographic heterogeneity underscores the need for tailored interventions based on local epidemiological patterns.

### 2.4 Risk Factors and Transmission Patterns

Several key risk factors drive hepatitis B transmission in Florida. Analysis of 2019 acute Hepatitis B data from the Florida Department of Health indicates that injection drug use accounted for 24% of hepatitis B cases within 6 months of infection, followed by non‐injection drug use (22%) and incarceration for over 24 h (14%) [10]. These patterns highlight how drug use, injection or non‐injection based, and incarceration are major risk factors for contracting acute Hepatitis B in Florida.

## 3 Prevention and Public Health Response

### 3.1 Vaccination Coverage and Potential Concerns

Hepatitis B vaccination coverage in Florida closely aligns with national rates. Based on data from the CDC's ChildVaxView tool, an estimated 92.9% of individuals in Florida's 2020–2021 birth cohort received at least three doses of the hepatitis B vaccine, compared to 92.6% at the national level [11].

However, a potential emerging challenge is the decline in vaccination rates, driven in part by the increasing spread of vaccine misinformation following the COVID‐19 pandemic [12]. According to the Florida Department of Health, the percentage of fully immunised two‐year‐olds declined from 86.7% in 2013 to 76.6% in 2022 [13]. Additionally, religious exemptions for school‐aged children (ages 5–17) continue to rise, with a statewide prevalence of 6.35% as of March 31, 2025 [14]. While current hepatitis B vaccination rates do not indicate significant health disparities compared to national figures, these trends present concerning implications for future vaccination coverage and public health outcomes.

These trends are particularly concerning considering recent statements by Governor Ron DeSantis and State Surgeon General Joseph Ladapo. On September 3rd 2025, during a press conference, Dr. Ladapo announced his intent to eliminate all vaccine requirements and urged the state legislature to enact corresponding policy changes [15]. Notably, certain immunisation requirements—such as those for hepatitis B—are not codified in state law and are reportedly in the process of being rescinded [15]. This development is deeply troubling, as school‐entry immunization mandates constitute a cornerstone of public health infrastructure in the United States [16]. The removal of such requirements would likely precipitate a sharp decline in vaccination coverage and substantially increase the risk of vaccine‐preventable disease outbreaks [16].

### 3.2 Perinatal Hepatitis B Prevention Program

Florida's Perinatal Hepatitis B Prevention Program (PHBPP) represents one of the state's most successful public health interventions against HBV. The program conducts routine HBsAg screening for all pregnant women and provides follow‐up care for those who test positive [17]. For exposed infants, the program ensures timely delivery of post‐exposure prophylaxis, including hepatitis B immune globulin (HBIG) and hepatitis B vaccines administered within 12 h of birth, followed by additional doses at 1 and 6 months [17].

The effectiveness of this approach has been successful, with only one case reported between 2020 and 2023 and eight cases documented from 2013 to 2023 [18]. While these numbers are low, perinatal hepatitis B remains rare, with just 7 cases reported nationwide in 2023, according to the CDC [19]. Nevertheless, this program plays a critical role in improving health outcomes for newborns born to hepatitis B‐positive mothers.

### 3.3 Adult Vaccination and Testing Program

The Florida Hepatitis Prevention Program (HPP) funds the Adult Vaccination and Testing Program in 15 counties: Alachua, Bay, Broward, Collier, Duval, Escambia, Lee, Miami‐Dade, Monroe, Okeechobee, Orange, Palm Beach, Pinellas, Polk and Seminole [20]. This program offers hepatitis A and B immunizations, screening for chronic hepatitis B and C, education, surveillance, targeted interventions and epidemiological investigations [20].

Despite these efforts, significant geographic disparities in service accessibility persist. The counties with the highest rates of acute hepatitis B (Jefferson and Levy) and chronic hepatitis B (Washington and Union) are not covered by the Adult Vaccination and Testing Program, highlighting a critical gap in the state's prevention strategy [4, 6, 20].

### 3.4 Harm Reduction and Co‐Morbidity

Florida's approach to harm reduction for hepatitis B prevention has been limited. The Infectious Disease Elimination Act (IDEA) program, which focuses on reducing the transmission of HIV, hepatitis B and hepatitis C through syringe exchange programs (SEPs), currently operates in only eight counties: Miami‐Dade, Broward, Palm Beach, Pinellas, Hillsborough, Orange, Alachua and Leon [21]. This leaves most Florida counties, particularly rural areas with high hepatitis B rates, without access to these critical harm reduction services [19].

Moreover, the IDEA program's emphasis has been predominantly on HIV and hepatitis C, with limited attention to hepatitis B. This is evidenced by the absence of hepatitis B data in the IDEA fact sheet and the lack of emphasis on hepatitis B testing or vaccinations at SEPs [22]. Also, the prohibition on using state, county, or municipal funds to support Syringe Exchange Programs (SEPs) in Florida severely limits their ability to expand and sustain operations [23]. As a result, these programs must rely entirely on private funding sources, such as donations and grants, which are often limited and inconsistent. This funding constraint hinders the scalability of SEPs, reducing their capacity to offer essential services like hepatitis B testing, vaccination referrals and harm reduction education, ultimately impacting their effectiveness in addressing the spread of infectious diseases [23].

There is also substantial co‐morbidity between Hepatitis B and HIV. In 2023, hepatitis B and HIV co‐infection affected 278 individuals, representing a comorbidity rate of 2.2% among people with HIV [8]. This underscores the need for integrated approaches to address overlapping diseases.

### 3.5 Testing Coverage and Barriers

Hepatitis B testing coverage in Florida remains inadequate, particularly among underserved populations. A recent study of over 91,000 patients at Florida Federally Qualified Health Centers found that only 43.7% had ever been screened for HBV, despite CDC recommendations for universal adult screening [24]. Substantial disparities in testing were observed, with Haitian Creole‐speaking (aOR 1.91) and Asian patients (aOR 1.50) having higher odds of screening, while non‐Hispanic White and English‐speaking Hispanic patients had notably lower rates [24]. Barriers to screening include limited provider adherence to updated guidelines, patient mistrust and lack of awareness and systemic issues such as inadequate insurance coverage—especially among Medicare recipients [24]. Addressing these gaps is critical to meeting national hepatitis elimination goals and improving health outcomes for at‐risk communities.

### 3.6 Educational Resources

The Florida Department of Health's Viral Hepatitis and Outbreak Response Section (VHORS) has attempted to address these gaps by offering healthcare workers two online training programs: Hepatitis 101, covering risk factors, symptoms, transmission, and vaccination and the Viral Hepatitis Serology Workshop, focusing on interpreting serological tests and distinguishing between acute and chronic infections [25]. However, the impact of these educational initiatives on testing practices remains unclear.

## 4 Progress Toward Elimination and Future Directions

### 4.1 Alignment With WHO Elimination Goals

In 2016, the World Health Organization established targets for viral hepatitis elimination by 2030, including a 90% reduction in new infections and a 65% reduction in mortality [26]. Florida's progress toward these goals has been mixed. Although Florida exhibits lower hepatitis B–related mortality rates compared to national trends, it continues to experience higher incidence rates of both acute and chronic hepatitis B infections relative to the national averages [1, 3, 7].

The increasing incidence of acute hepatitis B in Florida, contrasting with declining national trends, indicates that the state is not on track to meet the 90% reduction target for new infections. Similarly, current mortality rates from HBV‐related complications suggest insufficient progress toward the 65% mortality reduction goal.

### 4.2 Policy and Program Recommendations

To accelerate progress toward hepatitis B elimination in Florida, several strategic interventions are recommended:

1. Expanded Testing and Linkage to Care: Implementation of universal routine screening for all adults, in accordance with current CDC guidelines. Improved linkage systems to connect diagnosed individuals with appropriate care and treatment services.
2. Enhanced Harm Reduction Services: Expansion of syringe exchange programs beyond the current five counties, with explicit inclusion of hepatitis B testing and vaccination services. Removal of restrictions on funding sources for harm reduction programs to enable greater scalability and sustainability.
3. Targeted Vaccination Strategies: Development of innovative approaches to increase adult hepatitis B vaccination coverage, particularly among high‐risk groups and in rural counties with elevated acute infection rates.
4. Strengthened Healthcare Infrastructure: Investment in healthcare capacity, especially in underserved rural areas, including telehealth options for hepatitis specialty care and decentralised treatment delivery models.
5. Integrated Approaches: Implementation of comprehensive, integrated programs addressing viral hepatitis alongside related conditions such as HIV, substance use disorders and other sexually transmitted infections.
6. Enhanced Surveillance: Strengthening of the hepatitis surveillance system to provide more complete and timely data on the complete care cascade, including treatment initiation, adherence and outcomes.
7. Addressing Social Determinants: Development of interventions addressing the underlying social and structural factors contributing to hepatitis B disparities, including poverty, housing instability, stigma and discrimination.
8. Infant and Child Vaccination Coverage: Universal administration of the hepatitis B birth dose for all newborns; completion of the 3‐dose series by 24 months; and maintenance and enforcement of school‐entry immunisation requirements.

### 4.3 Research Priorities

Key research priorities for advancing hepatitis B control in Florida include:

1. Implementation of science studies to identify effective strategies for scaling up testing and treatment in diverse settings, particularly in underserved communities.
2. Epidemiological investigations to better understand transmission networks and risk factors in high‐incidence counties.
3. Health services research examining barriers and facilitators to care engagement across the hepatitis B care continuum.
4. Economic analyses to quantify the cost‐effectiveness of expanded prevention and treatment interventions, providing evidence to support policy decisions and resource allocation.
5. Evaluation studies assessing the impact of recent policy changes and program implementations on hepatitis B outcomes.
6. Behavioural and policy research exploring the underlying causes of waning vaccine uptake among infants and young children, including parental attitudes, misinformation, access barriers and the effects of changing immunisation requirements.

## 5 Conclusion

Hepatitis B remains a significant public health challenge in Florida, characterised by disparities in disease burden across geographical, demographic and socioeconomic lines. Despite some successful prevention efforts, particularly in preventing perinatal transmission, the state continues to experience higher rates of both acute and chronic hepatitis B compared to national averages.

Achieving the WHO's elimination goals by 2030 will require a multifaceted approach addressing gaps across the prevention and care continuum. This includes expanding access to vaccination, testing and treatment; strengthening surveillance systems; implementing effective harm reduction strategies; and addressing the social determinants driving hepatitis B disparities.

With concentrated effort and strategic investment, Florida can reverse current trends and make substantial progress toward eliminating hepatitis B. Success will require collaboration across sectors, engagement of affected communities and sustained commitment to evidence‐based interventions tailored to the state's diverse population and unique epidemiological context. However, considering recent policy shifts and the absence of school‐entry vaccination mandates, emerging gaps in immunisation coverage among younger populations could threaten these gains and pose significant long‐term public health consequences.

## Ethics Statement

Ethical review and approval were not required for this study as it involved the analysis of publicly available, de‐identified data obtained from the Florida Department of Health and the U.S. Centers for Disease Control and Prevention.

## Conflicts of Interest

The author declares no conflicts of interest.
